# Application of Supervised Machine Learning Techniques and Digital Image Analysis for Predicting Live Weight in Anadolu-T Broilers

**DOI:** 10.3390/ani16010068

**Published:** 2025-12-25

**Authors:** Erdem Küçüktopçu, Bilal Cemek, Didem Yıldırım, Halis Simsek, Kadir Erensoy, Musa Sarıca

**Affiliations:** 1 Department of Agricultural Structures and Irrigation, Ondokuz Mayıs University, Samsun 55139, Türkiye; bcemek@omu.edu.tr (B.C.); ydidem19@gmail.com (D.Y.); 2 Department of Agricultural and Biological Engineering, Purdue University, West Lafayette, IN 47907, USA; simsek@purdue.edu; 3 Department of Animal Science, Ondokuz Mayıs University, Samsun 55139, Türkiye; kadir.erensoy@omu.edu.tr (K.E.); msarica@omu.edu.tr (M.S.)

**Keywords:** poultry, artificial intelligence, morphometric traits, computer, pixel

## Abstract

Accurate estimation of live weight is essential for efficient management and precision control in poultry production. This study evaluated the potential of supervised machine learning (ML) algorithms and digital image analysis for non-invasive prediction of live weight in Anadolu-T broilers, a locally developed genotype in Türkiye. A total of 4200 records were collected from 100 broilers (50 males and 50 females) over 42 days, including daily measurements of back length, back width, and live weight. Five ML algorithms—Random Forest (RF), k-Nearest Neighbors (KNN), Support Vector Regression (SVR), Extreme Gradient Boosting (XGB), and Multiple Linear Regression (MLR)—were trained and validated to estimate live weight based on morphometric traits. Among all algorithms, KNN achieved the highest accuracy (R^2^ = 0.982, RMSE = 111.509 g, MAPE = 8.205%), followed by RF and XGB, which also produced stable and reliable predictions. The image-based models using log-transformed regression between body surface pixel area and live weight yielded similar accuracy (R^2^ = 0.989, RMSE = 101.197 g, MAPE = 7.266%), confirming that projected surface area can effectively represent growth progression. The results demonstrate that integrating ML algorithms with digital imaging offers a practical, cost-effective, and non-invasive approach for real-time broiler weight estimation. This approach supports the advancement of precision poultry farming through automated, data-driven growth monitoring.

## Simple Summary

Measuring the live weight of chickens is essential for proper feeding, health monitoring, and production planning on poultry farms. However, manual weighing of chickens can be time-consuming for farmers. This study investigated the use of computer-based methods and digital image analysis to estimate the live weight of broiler chickens without the need for direct weighing. In computer-based methods, the weight of broiler chickens was estimated using simple body measurements, such as the length and width of the back. In digital image analysis, the number of body surface pixels and the day of age were used to predict live weight. The results showed that both computer-based methods and digital image–based models provided highly accurate predictions, whereas traditional linear methods were less effective. These findings demonstrate that modern computer vision and machine learning tools can offer fast, reliable, and non-invasive alternatives for monitoring broiler growth. This approach supports smarter and more sustainable practices in precision poultry farming.

## 1. Introduction

Global meat consumption is expected to increase significantly in the coming decades due to the growing world population and increasing demand for animal protein, especially in developing regions, where urbanization and rising income levels are rapidly changing dietary habits [1]. Of all livestock sectors, poultry stands out as the fastest growing, with both the production and consumption of poultry meat rising sharply worldwide [2]. This trend is especially pronounced in developing countries, which now account for the majority of global poultry meat production and have overtaken industrialized countries in recent decades [3]. The widespread appeal of poultry products stems from their affordability, efficient feed conversion, and short production cycles, which make them an important source of high-quality protein and essential nutrients for billions of people worldwide.

As poultry meat continues to play a central role in meeting global protein demands, improving production efficiency has become increasingly important for sustainable growth in the sector. In broiler farming, live weight is an important measure of growth efficiency, as it is crucial for producers to know both the average weight and the weight distribution within a flock at the time of slaughter [4].

Traditionally, to determine live weight, individual birds are manually caught and weighed using electronic or manual scales [5]. Although widely used, this method is labor-intensive, time-consuming, and can compromise animal welfare [6]. Automated weighing platforms have been introduced to alleviate these drawbacks by collecting weight data as birds move freely within the housing environment [7,8]. However, platform engagement can be inconsistent across the flock, as dominant or heavier birds may underutilize the system, resulting in biased or incomplete data [9]. These limitations have stimulated interest in alternative, non-invasive approaches to estimate live weight.

Given the limitations of both manual weighing and current automated systems, recent research has increasingly focused on developing predictive models that utilize easily obtainable phenotypic traits to estimate broiler live weight more efficiently. Morphometric characteristics, such as body length, width, and other linear measurements, have shown strong correlations with body weight and are therefore considered promising predictors for non-invasive weight estimation models [10,11,12]. For instance, the authors in [10] utilized 270 broiler chicks, consisting of 90 individuals from each of the Arbor Acres, Ross, and Marshall strains, to estimate live weight using body length and width measurements. Similarly, in the study by [13] investigated the repeatability of body weight and linear body traits in 100 Marshall broiler chickens from hatch to eight weeks of age, highlighting the consistency of these measurements over time. In another study, ref. [14] developed estimation equations based on morphometric traits to predict live weight specifically in Ross genotype broilers.

Building on these findings, advances in computer vision and digital imaging now offer an additional way to capture morphometric information without direct handling. Far from being confined to poultry, image analysis techniques have been used successfully applied many livestock species to estimate body weight, carcass composition or growth characteristics [15,16,17,18]. Despite this wide application, the integration of image-based measurements with predictive models in poultry is still limited, although it offers considerable potential to reduce labor input and to continuously monitor flocks.

At the same time, the poultry sector generates large volumes of data through its intensive production cycles, providing an ideal context for the application of supervised machine learning (ML) algorithms. Techniques such as artificial neural networks (ANN) [19,20], support vector regression (SVR) [21,22], random forests (RF) [23], and related ensemble methods have shown high predictive accuracy for broiler live weight in previous studies. Yet most of this work has focused narrowly on commercial strains such as Ross and Cobb [24,25,26,27], leaving open the question of whether similar predictive relationships hold for alternative or locally bred genotypes developed under different breeding strategies or regional conditions.

To address the aforementioned gaps in the literature, the present study aims to achieve the following objectives:

- Conduct a comprehensive comparative evaluation of multiple supervised ML algorithms, including RF, SVR, K-Nearest Neighbors (KNN), Extreme Gradient Boosting (XGB), and Multiple Linear Regression (MLR) to predict live weight in Anadolu-T broilers (a native Turkish genotype selected for fast-growth, feed efficiency and breast meat yield) [28,29] based on simple morphometric inputs (back length and width).
- Investigate potential gender effects by constructing separate models for male, female, and mixed-sex datasets.
- Explore a complementary digital image analysis approach to quantify broiler body surface area and derive a linear equation for estimating live weight from body-surface pixels.
- Establish the growth curve of Anadolu-T broilers using the daily live-weight records collected over the 42-day experimental period.

By integrating morphometric measurements, ML, digital imaging, and growth-curve modeling within a three-stage framework, this study extends non-invasive live-weight prediction to a regionally important broiler line and provides new insights for data-driven precision poultry farming.

## 2. Materials and Methods

**Figure 1:**
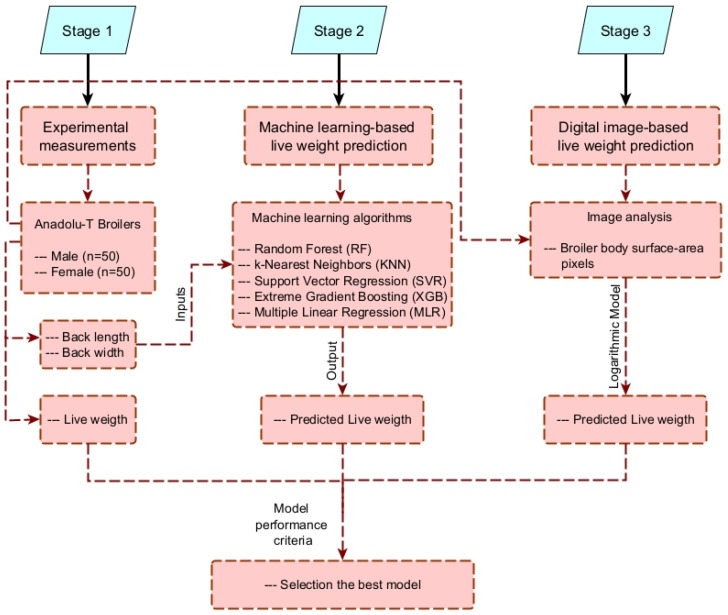
Systematic overview of the live weight prediction model.

The diagram (Figure 1) summarizes the three-stage workflow used in this study. In Stage 1, daily experimental measurements were collected from Anadolu-T broilers, including back length, back width, and live weight. In Stage 2, these morphometric traits served as inputs for five ML algorithms (RF, KNN, SVR, XGB, and MLR) to estimate live weight. In Stage 3, a complementary digital image-based approach was implemented, in which surface-area pixels extracted from top-view photographs were used to predict live weight through a logarithmic model. The predicted values obtained from both approaches were then evaluated using performance metrics to determine the most accurate method.

### 2.1. Study Area and Husbandry Conditions

**Figure 2:**
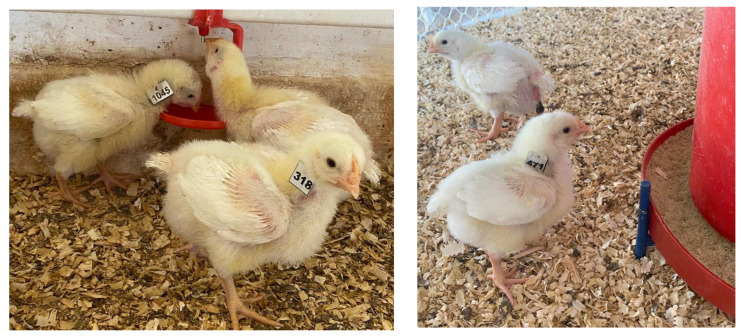
Representative view of one-day-old Anadolu-T chicks.

The study was conducted at the Research and Application Farm of the Faculty of Agriculture, Ondokuz Mayıs University, located in Samsun, Türkiye (41°21′ N, 36°11′ E). The experiment was carried out in an environmentally controlled poultry house, where the birds were housed in experimental pens measuring 2.2 × 2.2 m with a height of 0.5 m, separated by wire mesh partitions. The experimental population consisted of 100 one-day-old Anadolu-T broiler chickens (Figure 2), including 50 males and 50 females. To eliminate potential sex-related behavioral effects and ensure consistent data collection, male and female chicks were housed in separate pens.

All pens were bedded with 8–10 cm of wood-shaving litter, which was inspected daily and refreshed as necessary to prevent excessive moisture buildup. Stocking density was maintained at approximately 10 birds m^−2^, in accordance with standard broiler production recommendations. A 23L:1D lighting schedule was applied during the first week to promote early feed intake and thermoregulation, followed by a 20L:4D photoperiod for the remainder of the experiment. Birds were provided a three-phase commercial broiler diet (starter, grower, and finisher), with feed and water offered ad libitum. Routine sanitary procedures—including daily removal of wet litter patches, weekly disinfection of feeders and drinkers, and continuous monitoring of air quality—were implemented uniformly across all pens.

### 2.2. Experimental Measurements

The experiment lasted six weeks (42 consecutive days). Throughout this period, the live weight, back length, and back width of each bird were measured individually on a daily basis to ensure continuous monitoring of growth and morphometric development. Back length was measured from the cranial tip of the scapula to the posterior end of the synsacrum, and back width was measured at the widest lateral point between the left and right thoracic regions using a rigid 300 mm ruler (±1 mm precision). Each bird was gently positioned in a natural standing posture on a flat surface, and all measurements were collected by the same trained personnel to minimize operator-related variability.

In total, 4200 individual records were obtained (100 birds × 42 days), providing sufficient statistical power and robustness for training and validating the ML models. By capturing temporal growth dynamics on a daily basis, the dataset reflects both inter-individual variability (differences between birds) and intra-individual growth trajectories (daily changes within the same bird), thereby strengthening the predictive modeling process. However, because these records represent repeated daily measurements from the same individuals rather than independent samples, the observations cannot be considered fully independent. For the ML analysis, the data were randomly split at the record level into a training set (80%) and a testing set (20%), so that different days from the same bird could appear in both sets. Consequently, the reported performance metrics should be interpreted within this repeated-measures time-series framework, rather than as predictive performance on entirely independent populations.

### 2.3. Growth Curve Modeling Approach

The live weight data of broilers were modeled using the Richards function [30] a flexible sigmoidal growth model widely applied in animal growth studies [31,32,33]. This model was selected because it can accurately represent the nonlinear and asymptotic nature of biological growth, allowing for the estimation of parameters that describe both the growth rate and the inflection point of the curve. The mathematical form of the Richards function is expressed as:

(1) $$LW={A(1+Be^{-kt})}^{-1/m}$$

where *LW* is the live weight (g), *A* is the asymptotic maximum weight, *B* is an integration constant related to the initial condition, *k* is the growth rate constant, *t* is the age in days, and *m* is a dimensionless shape parameter that determines the position of the inflection point.

### 2.4. Machine Learning-Based Live Weigth Prediction

In this study, five ML algorithms (RF, XGB, SVR, KNN, and MLR) were selected as baseline models to predict the live weight of broilers. These algorithms were chosen based on their different learning strategies, their robustness to different data structures, and their proven effectiveness in previous studies related to livestock performance prediction [34,35,36,37]. RF and XGB, as ensemble-based tree algorithms, are well-suited to handle nonlinear relationships and can effectively manage high-dimensional feature spaces and potential interactions between variables [38,39,40]. SVR is a kernel-based method that performs well in small to medium-sized datasets and is particularly effective in capturing complex, non-linear patterns with controlled overfitting [41,42,43]. KNN, a non-parametric, instance-based learner, offers simplicity, and adaptability to different data distributions without requiring prior assumptions [44,45,46]. Lastly, MLR was included as a classical benchmark model to evaluate the relative performance improvements offered by more advanced, non-linear algorithms [47,48,49].

Although the feature set in this study consists of only two morphometric predictors, these inputs exhibit inherently nonlinear relationships with live weight. Therefore, nonlinear ML algorithms were chosen because they can flexibly capture complex biological patterns without imposing fixed functional structures. MLR was deliberately included as a classical baseline, enabling direct comparison between simple linear modeling and more flexible ML approaches.

To evaluate the effect of gender on model performance, three different datasets were constructed:

Model 1: a dataset consisting exclusively of female broiler data,
Model 2: a dataset containing only male broiler data, and
Model 3: a combined dataset including both male and female data.

For model development, the overall dataset was randomly divided into a training set (80%) and a testing set (20%). From the training set, 90% was further allocated as the primary training subset and used for hyperparameter tuning through internal cross-validation. This process was repeated 10 times to ensure robustness and reliability of the evaluation results, following a repeated random sub-sampling validation approach [50]. Prior to model training, all input features were standardized to a range between 0 and 1 to minimize scale-related bias and enhance model convergence. The entire analysis was carried out on a 64-bit Windows 11 operating system using a personal computer equipped with an AMD Ryzen 7 CPU (3.2 GHz) and 16 GB of RAM, which provided sufficient computational efficiency for iterative training and testing of the ML models.

### 2.5. Digital Image-Based Live Weigth Prediction

For image acquisition and weight data collection, a custom-designed platform was constructed to hold each bird individually while photographs were taken. The platform had a black floor (600 × 400 mm), providing a clear contrast background that enhanced the visibility of the bird’s outline. The images were captured using the digital camera of a smartphone (iPhone 14, Apple Inc., Cupertino, CA, USA). The device was mounted centrally above the bird at a fixed height of 1.0 m. This distance was kept constant throughout the experiment, thereby ensuring that all images were captured at a uniform scale. To promote posture consistency, each bird was placed on the non-slip platform in a natural standing position, and photographs were taken within a few seconds to minimize any posture-related variation. Moreover, five consecutive images were captured for each bird, and the mean pixel value was used to reduce noise arising from minor differences in posture. The accuracy of broiler body surface estimation largely depends on the quality of the acquired images. To minimize the potential influence of image noise, the pixel value for each bird was calculated as the mean of five replicated digital images.

**Figure 3:**
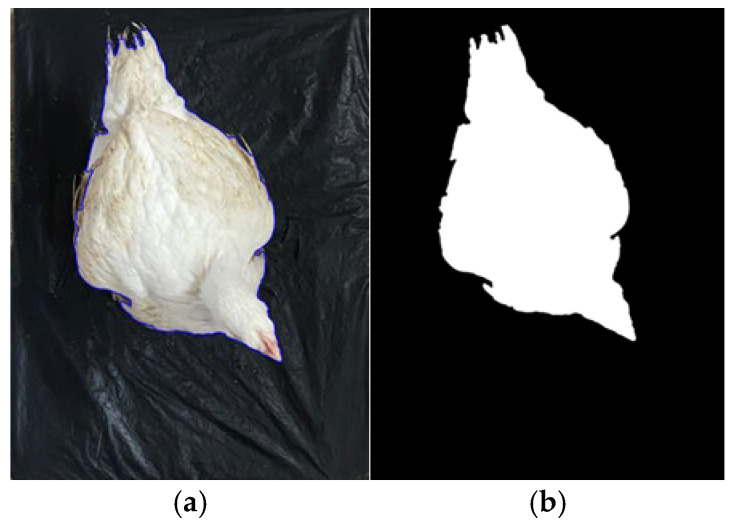
Illustration of the image-processing workflow. (**a**) Top-view image of a broiler placed on the black reference floor with its body boundaries outlined, (**b**) Binary mask of the bird obtained after segmentation.

A fully automated computer-vision workflow was implemented in Python (Python Software Foundation, Wilmington, DE, USA) to measure the projected pixel area of individual broilers from top-view images. The pipeline consisted of four major steps: (i) detection of the calibrated region of interest (ROI) defined by the black reference floor, (ii) segmentation of the bird within the ROI, (iii) pixel counting of the foreground region corresponding to the bird’s body, and (iv) export of per-image measurements and quality-control overlays (Figure 3). Subsequently, the extracted pixel counts were directly regressed against the corresponding live weight records of the birds to develop predictive models for digital image-based weight estimation.

For consistency with the ML-based approach, the image-based regression models were evaluated using the same train–test split (80% training and 20% testing) as in the ML analyses. After extracting the pixel-derived predictors, the image-based equations were fitted using the training subset and then evaluated on the same independent test subset. This ensured that all predictive approaches (ML models and image-based regression) were assessed under the same data partitioning scheme, providing fully comparable performance metrics.

### 2.6. Model Performance Criteria

In this study, the coefficient of determination (R^2^), the root mean square error (RMSE), and the mean absolute percentage error (MAPE) and were employ.

(2) $$R^{2}=1-\frac{\sum_{i=1}^{n}{\left(y_{mea}-y_{pre}\right)}^{2}}{\sum_{i=1}^{n}{\left(y_{mea}-y_{avg}\right)}^{2}}$$

(3) $$RMSE=\sqrt{\frac{1}{n}\sum_{i=1}^{n}{\left(y_{mea}-y_{pre}\right)}^{2}}$$

(4) $$MAPE=\frac{1}{n}\sum_{i=1}^{n}\left(\frac{\left|y_{mea}-y_{pre}\right|}{y_{mea}}\right)\times 100$$

where *y_mea_* denotes the actual value, *y_pre_* symbolizes the predicted value, *y_avg_* is the average actual value, and *n* is the total data number.

## 3. Results

### 3.1. Environmental Conditions

**Figure 4:**
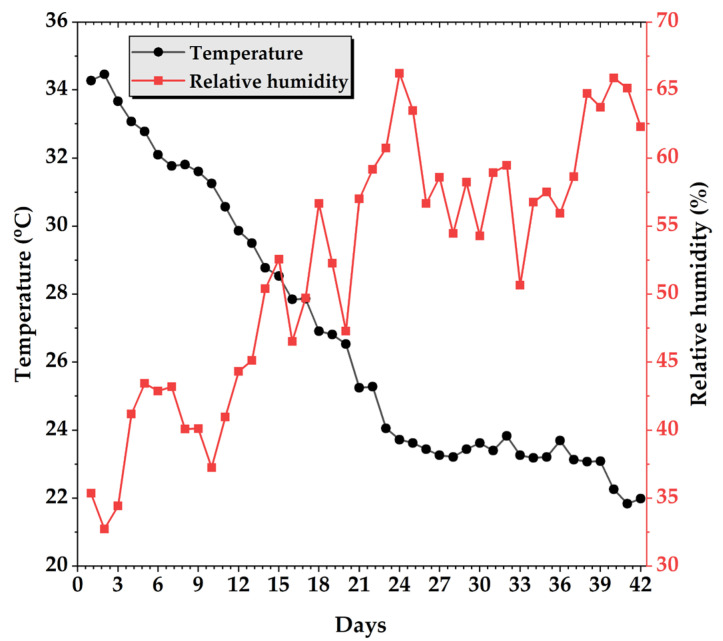
Variation in temperature (°C) and relative humidity (%) inside the poultry house during the 42-day rearing period.

Temperature and relative humidity inside the poultry house were continuously monitored throughout the 42-day rearing period in order to maintain stable environmental conditions for the experimental birds. At the beginning of the experiment, the mean indoor temperature was approximately 34 °C, which gradually decreased to about 22 °C by the end of the trial (Figure 4). Conversely, relative humidity exhibited an increasing trend, rising from about 20% at the start to approximately 65% towards the end of the period. Such changes reflect the combined effect of controlled ventilation and the natural accumulation of moisture as bird biomass and litter moisture increased over time. Throughout the trial, both parameters remained within the recommended ranges for broiler production [51,52,53] indicating that the rearing environment was adequately controlled and did not introduce confounding effects on growth performance or morphometric measurements.

### 3.2. Growth Performance of Broilers

**Figure 5:**
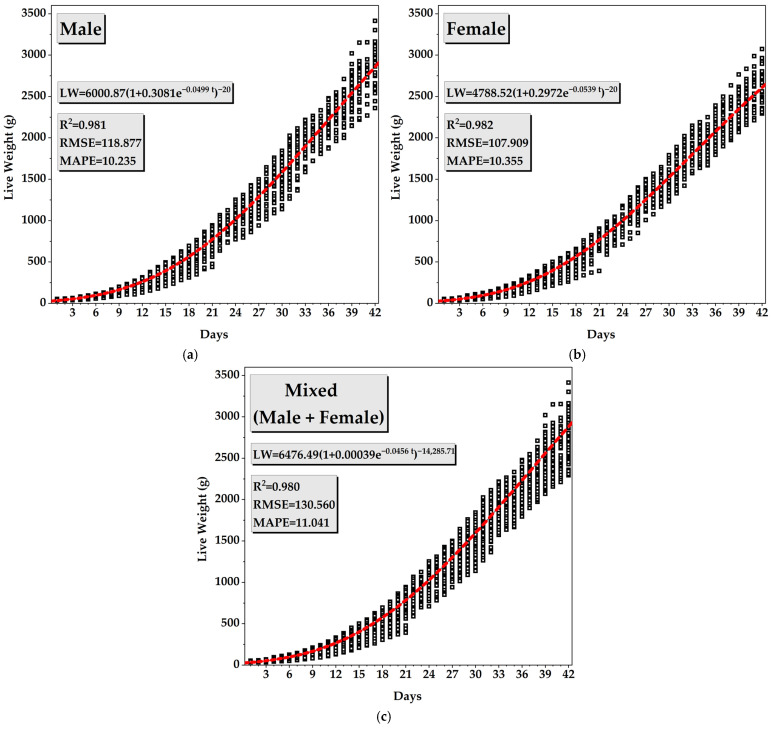
Growth curves of broiler live weight over the 42-day rearing period for (**a**) male, (**b**) female, and (**c**) mixed groups (In the equations, *LW* denotes live weight (g), and *t* represents days).

The growth performance of broilers is illustrated in Figure 5 for male, female, and mixed (male + female) groups. In all groups, live weight increased progressively over the 42-day rearing period, following a characteristic sigmoidal growth pattern typical of broiler development. The initial live weight was approximately 30 g for all groups, with growth accelerating notably after the second week and continuing until the sixth week, when the birds approached market age.

As shown in Figure 5a–c, male broilers consistently exhibited higher live weights compared to females throughout the experiment. According to the fitted Richards function, males reached an asymptotic live weight of 6000.87 g, demonstrating a high degree of model accuracy (R^2^ = 0.981, RMSE = 118.877 g, MAPE = 10.235%). Females attained a lower asymptotic live weight of 4788.52 g, but the model fit remained equally strong (R^2^ = 0.982, RMSE = 107.909 g, MAPE = 10.355%,). The mixed group exhibited intermediate growth characteristics, with an asymptotic live weight of 6476.49 g, and a satisfactory model performance (R^2^ = 0.980, RMSE = 130.560 g, MAPE = 11.041%).

The fitted Richards curves provided an excellent fit to the observed live weight data, successfully describing the sigmoidal growth pattern typical of broiler development. The model captured the gradual increase during the early phase, the rapid growth period between the second and sixth weeks, and the final asymptotic stage as the birds approached market age. The R^2^ and low prediction errors (MAPE and RMSE) confirmed the strong agreement between predicted and observed values, demonstrating the robustness of the model in representing biological growth processes [54].

Sex had a clear influence on the estimated growth parameters. Male broilers exhibited higher asymptotic body weights and faster growth rates than females, consistent with their larger mature body size and greater feed utilization efficiency. In contrast, females showed a more moderate growth trajectory, while the mixed group displayed intermediate characteristics, reflecting the natural biological variability of both sexes. Overall, the Richards model accurately captured these differences, providing a reliable framework for evaluating growth dynamics and performance potential in broilers under commercial rearing conditions.

### 3.3. Descriptive Statistics of Experimental Data

The descriptive statistics of the input variables (back length and width) and the output variable (live weight) are summarized in Table 1 for male, female, and mixed groups. In all groups, live weight increased from ~30 g at the start of the experiment to over 3000 g by the end, with mean values around 1000 g. Back length averaged 143–144 mm and width 104–105 mm across sexes, showing consistent patterns. Overall, the distributions of live weight and morphometric traits were slightly skewed and relatively flat, indicating comparable growth trends among males, females, and the mixed dataset.

### 3.4. Performance Evaluation of ML Algorithms

The ML algorithms were employed to predict broiler live weight from morphometric traits, aiming to achieve higher accuracy than conventional linear regression. To maximize predictive performance, particular attention was given to hyperparameter optimization, which plays a decisive role in determining the learning capacity and generalization ability of ML models.

In this study, the hyperparameters of all employed algorithms (RF, SVR, KNN, and XGB) were optimized using a grid search approach combined with k-fold cross-validation on the training dataset. This procedure aimed to determine the optimal hyperparameter combinations that minimize predictive error and improve model generalization. After the optimal configurations were identified, each model was retrained on the entire training dataset and subsequently evaluated on an independent testing set. This strategy ensures that the hyperparameter tuning process remains unbiased with respect to the testing data and allows for a more reliable assessment of model performance on unseen samples. The final hyperparameter settings used for each model are summarized in Table 2, providing a clear reference for the algorithm configurations adopted in this study.

The performance metrics of five different algorithms (RF, KNN, SVR, XGB, and MLR) were evaluated across three datasets: male, female, and mixed (Table 3). The evaluation was based on the R^2^, RMSE, and MAPE for both training and testing dataset.

In the male dataset, ensemble models (RF and XGB) demonstrated superior performance, achieving R^2^ values above 0.985 in testing with low RMSE (111–113 g) and MAPE (9%). Among them, XGB yielded the lowest training RMSE (83.056 g) and MAPE (7.769%), indicating its effectiveness in capturing complex nonlinear relationships between morphometric traits and live weight and its strong ability to generalize to unseen data. SVR and KNN also performed well, maintaining R^2^ = 0.985 in testing, whereas MLR exhibited considerably weaker predictive capability (R^2^ = 0.930) and much higher error levels (RMSE = 241.435 g, MAPE = 15.412%), confirming the limited suitability of linear models for this problem.

For female broilers, similar performance patterns were observed. RF and XGB again achieved the highest accuracies (R^2^ = 0.984) in testing, with XGB providing the lowest training errors (RMSE = 81.362 g, MAPE = 6.707%). SVR and KNN followed closely, showing nearly identical testing accuracies (R^2^ = 0.984) with slightly higher RMSE values (102–103 g) and MAPE around 8%. MLR once more exhibited inferior accuracy (R^2^ = 0.940, RMSE = 199.012 g, MAPE = 13.377%).

In the mixed-sex dataset, ensemble models maintained their dominance. XGB achieved the best testing performance (R^2^ = 0.984, RMSE = 106.688 g, MAPE = 9.133%), followed closely by RF (R^2^ = 0.983, RMSE = 107.378 g, MAPE = 8.853%). KNN and SVR also produced stable predictions with moderate error levels, while MLR remained the least effective (R^2^ = 0.942, RMSE = 200.305 g, MAPE = 13.909%).

**Figure 6:**
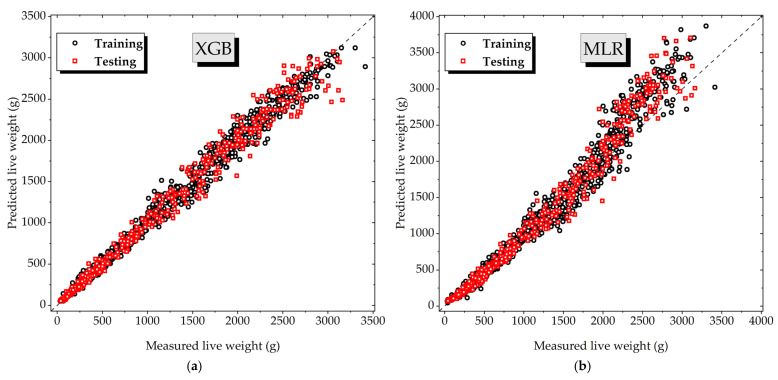

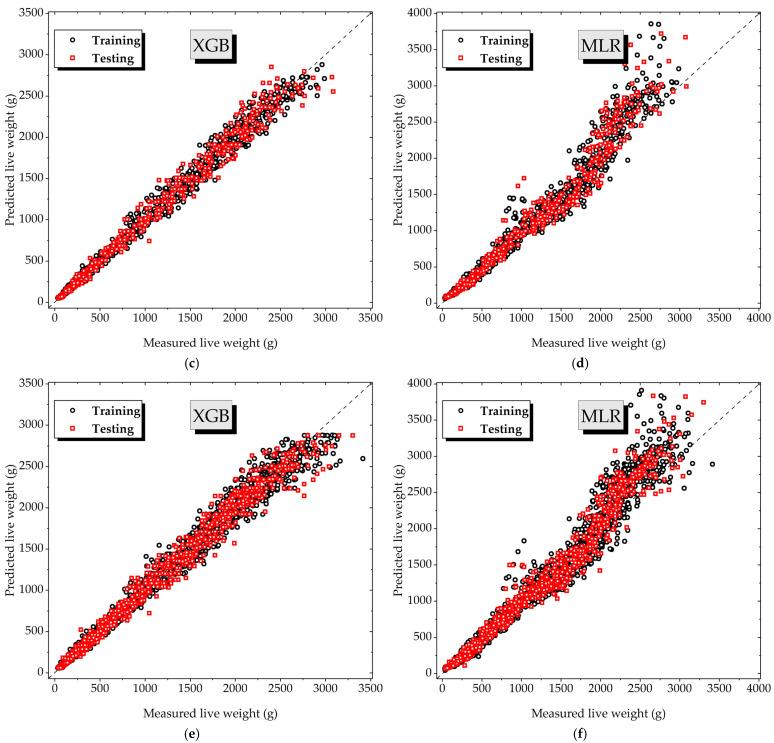
Scatter plots of measured versus estimated live weight values obtained using the XGB (best-performing) and MLR (worst-performing) models for the male (**a**,**b**), female (**c**,**d**), and mixed-sex (**e**,**f**) broiler datasets.

Across all three datasets (male, female, and mixed-sex), the XGB algorithm consistently produced the most accurate predictions, as evidenced by its high R^2^ values and low error metrics. In contrast, MLR exhibited the weakest performance, with substantially lower predictive accuracy and higher error values. To highlight these differences, the distribution plots of predicted versus observed live weights for both XGB (best-performing) and MLR (worst-performing) models are presented in Figure 6.

The scatter plots of the predicted versus measured live weights show that the estimates generated by XGB are closely aligned with the 1:1 reference line, confirming high predictive accuracy and minimal bias. A slight tendency toward overfitting is noticeable in the XGB model, particularly at higher weight ranges where the scatter of points becomes marginally wider; however, these deviations remain limited and do not significantly affect overall accuracy. By contrast, MLR displays the widest spread and more systematic deviations, especially at higher weights. This outcome underscores the inability of the linear model to capture the complex, non-linear relationships between morphometric traits and body weight, thereby reaffirming the superior robustness and generalization capacity of ensemble-based approaches such as XGB.

The influence of input features on model predictions was investigated using SHapley Additive exPlanations (SHAP), which provide consistent and locally accurate attributions for each predictor. This approach enabled a transparent interpretation of feature contributions across different ML algorithms and sex-specific datasets.

**Figure 7:**
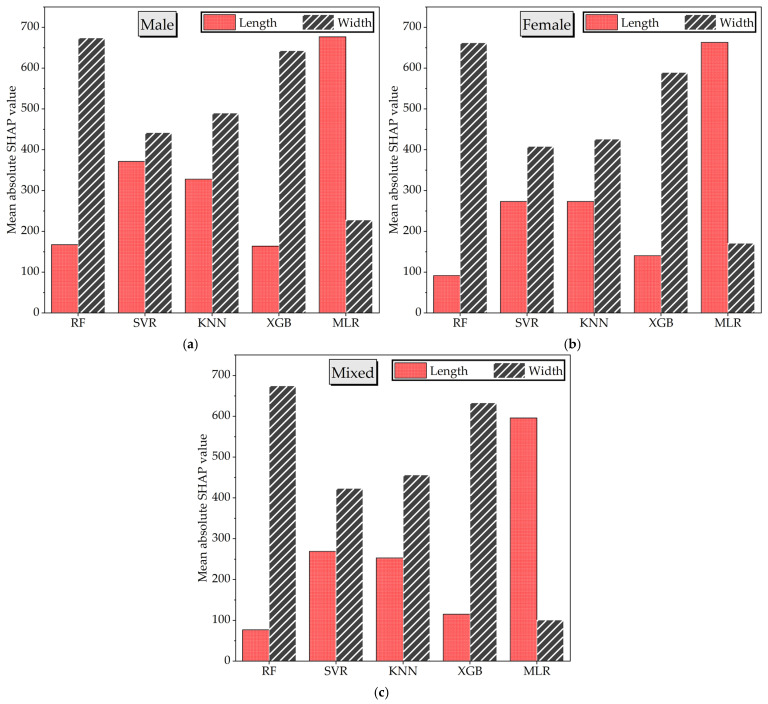
Feature importance, evaluated using the mean SHAP values by using the ML models for (**a**) male, (**b**) female and (**c**) mixed-sex broiler dataset.

The SHAP analysis revealed that the relative importance of length and width varied substantially across models and datasets (Figure 7). Overall, width emerged as the dominant predictor in most nonlinear models, particularly RF and XGB, where its contribution far exceeded that of length in both male and female groups. In contrast, MLR emphasized length over width, reflecting the sensitivity of linear models to direct relationships. KNN and SVR produced more balanced contributions, indicating that both features jointly supported the predictions. The mixed dataset largely reflected the same patterns observed in the sex-specific groups.

These results demonstrate that feature importance is highly model- and context-dependent: width consistently dominated in ensemble approaches, while length gained greater significance in linear contexts. Such variability underscores the necessity of subgroup-aware modeling when interpreting predictor contributions.

### 3.5. Performance Evaluation of Digital Image-Based Models

The predictive performance of the digital image–based equations was evaluated separately for male, female, and mixed (male + female) groups using the log-transformed relationships between body surface pixel number and live weight. Initially, a linear regression model was fitted between body weight and body surface area pixels. However, because both variables were repeatedly measured on the same birds across the 42-day growth period, temporal autocorrelation may exist in the data [15]. The log transformation was therefore applied not as a method to remove autocorrelation, but rather to stabilize variance and improve linearity in the relationship between pixel count and live weight.

Importantly, all performance metrics for the image-based models were obtained using the same independent test subset as used for the ML algorithms, ensuring direct comparability between the two approaches.

The fitted regression equations demonstrated high predictive accuracy for all datasets, with R^2^ values exceeding 0.98 (Table 4). The regression coefficients indicated that both Day and log P (log-transformed body surface pixel number) had strong and positive effects on log LW (log-transformed live weight), while the interaction term (Day × log P) had a small but significant negative effect, suggesting a slight reduction in the marginal effect of log P at later growth stages. Among the groups, the male model demonstrated excellent predictive capability, with R^2^ values of 0.987 (training) and 0.990 (testing), accompanied by low RMSE values (94.472–95.601 g). The female model produced similarly high R^2^ values (0.987–0.988) but achieved the lowest error rates overall, with RMSE values below 92.500 g and the smallest MAPE percentages (6.701–6.816%), indicating that the model performed with the greatest precision in this group. The mixed-sex model yielded intermediate results (R^2^ = 0.986–0.989; RMSE = 96.143–101.197 g), which is expected given the greater biological variability in the combined dataset. Overall, in all models, Day and log P maintained strong positive effects on log LW, while the interaction term consistently remained small yet significantly negative, reflecting a modest reduction in the marginal effect of log P as birds advanced in age.

**Figure 8:**
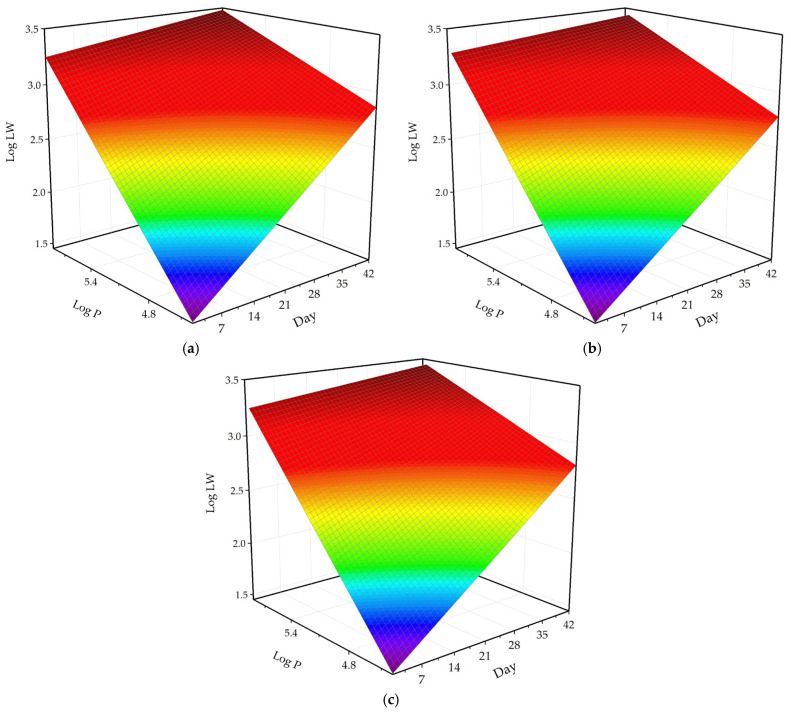
Three-dimensional response surfaces illustrating the relationships between log-transformed live weight (Log LW), log-transformed body surface pixel number (log P), and age (Day) for (**a**) male, (**b**) female, and (**c**) mixed (male + female) broiler groups.

The three-dimensional response surfaces (Figure 8) illustrate the relationship between log LW, log P, and Day for the male, female, and mixed datasets. In all groups, both log P and Day had strong positive effects on log LW, indicating that broiler live weight increased consistently with surface expansion and age progression. At early growth stages, the lower portions of the surfaces (blue–green regions) corresponded to low log LW values, while the gradient became steeper toward the later stages (yellow–red regions), reflecting the accelerated weight gain of broilers over time. The male model (Figure 8a) exhibited the steepest surface and highest log LW values, demonstrating its superior growth rate and higher sensitivity of body weight to both age and surface area. In contrast, the female model (Figure 8b) showed a slightly flatter surface, suggesting slower mass accumulation and a more stable, quasi-linear response. The mixed model (Figure 8c) presented intermediate behavior between males and females, confirming that it effectively generalized the relationship between image-derived pixel information and live weight across the population. Overall, the smooth and uniform color transitions across all surfaces confirmed that the log-transformed regression equations accurately captured the curvilinear nature of broiler growth and successfully represented the combined effects of surface expansion and age throughout the 42-day rearing period.

Preliminary analyses confirmed that all models exhibited strong linear associations (R^2^ = 0.98–0.99) across the growing period. Therefore, for simplicity, only RMSE and MAPE were presented to highlight the week-by-week variations in prediction accuracy (Table 5). These indices directly represent the absolute and relative deviation between observed and predicted live weights, providing an intuitive measure of the model’s practical performance. The weekly trend of RMSE and MAPE values indicates that the prediction accuracy of the log-transformed image-based model evolved systematically throughout the 42-day growing period. During the early growth stage (Days 1–14), both RMSE and MAPE values were relatively low across all datasets, confirming that the model accurately estimated live weight when the broilers were small and morphologically uniform. In the middle stage (Days 15–28), RMSE increased noticeably as body size and variability expanded, while MAPE remained stable around 7–8%, suggesting that although absolute errors grew with weight magnitude, the relative prediction accuracy remained consistent. In the final phase (Days 29–42), RMSE values continued to rise due to the wider range of live weights and individual growth heterogeneity, but MAPE decreased to approximately 5–6%, indicating that the model maintained robust proportional accuracy despite increasing dispersion. Overall, these results demonstrate that the image-based regression model successfully adapted to the dynamic growth process of broilers, providing stable and reliable performance across different age periods and population groups (male, female, and mixed).

## 4. Discussion

The findings of this study highlight the promising potential of supervised ML algorithms and digital image analysis as non-invasive tools for estimating live weight in Anadolu-T broilers. The consistently high prediction accuracy obtained across all datasets (R^2^ > 0.98) confirms that simple morphometric traits (particularly back length and width) contain sufficient biometric information to model body weight dynamics throughout the growing period.

Among the tested algorithms, the KNN model produced the most stable and accurate predictions, followed closely by ensemble-based approaches such as RF and XGB. The superiority of KNN may be attributed to its instance-based learning mechanism, which captures localized nonlinearities between morphometric traits and live weight without assuming an explicit functional form. Similar findings were reported different studies [55,56,57] where KNN outperformed more complex algorithms when the feature set was small and strongly correlated with body mass.

While ensemble models such as RF and XGB also performed well (especially in training scenarios) they showed slight signs of overfitting when evaluated with unseen test data. This is a common problem with ensemble methods, especially when tuning is not sufficiently regularized [58,59]. Nevertheless, their R^2^ values remained consistently high, indicating strong generalization. XGB often achieved the best training values with its regularized gradient boosting approach, but was narrowly outperformed by KNN and RF in testing phases.

SVR provided stable performance across all data sets, although it lagged slightly behind KNN and ensemble models in terms of RMSE and MAE. The ability of SVR to manage non-linear patterns through kernel functions was evident. However, the reliance on careful parameter tuning may have limited the fitting ability for all datasets equally [60,61].

The MLR, which was used as the base model, performed significantly worse in all scenarios. The rigid assumption of linear relationships and the susceptibility to multicollinearity probably contributed to higher prediction errors and lower R^2^ values. This emphasizes the limitations of classical regression models in capturing the complex biological variability inherent in phenotypic livestock data [62,63].

A major strength of this study is the use of SHAP analysis to interpret feature importance. The results revealed that back width was generally a more influential predictor than back length, especially in non-linear models like RF and XGB. In contrast, MLR models relied disproportionately on length, likely due to the linear structure favoring the most variance-explaining feature. This feature sensitivity demonstrates that even simple morphometric measurements can provide valuable predictive insights when interpreted by appropriate models.

The supplementary image-based modeling further validated the potential of digital vision as an alternative to manual measurements. The log-transformed regression equations exhibited strong linearity (R^2^ > 0.98) between body surface pixel area and live weight, confirming that projected surface area can reliably reflect growth progression. The weekly RMSE–MAPE trends indicated that the model adapted well to the broilers’ dynamic development, with proportional errors decreasing in later stages as birds attained higher weights. These findings corroborate earlier studies [15,20,25] demonstrating that properly calibrated imaging systems can provide accurate weight predictions for broilers and other livestock species.

Another valuable contribution of this research is the focus on Anadolu-T, a locally bred Turkish genotype, rather than commercial varieties such as Ross or Cobb. The genetic specificity and the novelty of this genotype make this breed a suitable candidate for testing the generalizability of ML approaches. The study thus extends the genetic and geographical scope of the existing literature and underlines that ML tools can also be effective beyond industrialized production systems.

From a practical perspective, implementing such models under field conditions could significantly reduce the need for error-prone automated platforms. These models could be integrated into low-cost digital systems that rely on simple measurements, allowing farmers to obtain near real-time estimates of live weight and growth efficiency.

Despite these promising outcomes, several limitations require explicit consideration. The dataset, while temporally dense, represents a relatively homogeneous group of 100 birds raised under identical, controlled environmental and management conditions. As such, the high predictive accuracies largely reflect intra-individual consistency rather than true population-level generalization across diverse production settings. In addition, all images were captured under highly standardized lighting, background, and camera positions. Although this controlled setup was necessary to evaluate baseline model performance, it does not fully reflect the variability present in commercial broiler houses, where inconsistent illumination, bird movement, flock density, and litter conditions may influence image quality and model robustness. A further limitation is the absence of independent external validation. Because train–test splitting occurred at the record level, measurements from the same individual could appear in both sets, meaning that the reported metrics should not be interpreted as predictive performance on unrelated populations. Future studies should therefore extend data collection across multiple flocks, genotypes, housing systems, and environmental contexts, while incorporating independent validation datasets and real-world imaging conditions to ensure broader applicability.

Overall, this study demonstrates that integrating morphometric measurements with supervised ML algorithms and digital imaging can provide an effective and biologically interpretable framework for real-time, non-contact monitoring of broiler growth. The strong predictive performance and physiological relevance of these models point to their potential as practical tools for precision poultry farming. However, the limitations noted above underscore the need for continued research that evaluates model robustness under commercial conditions and across diverse populations to fully realize the practical utility of these approaches.

## 5. Conclusions

This study demonstrated that supervised ML algorithms and digital image analysis can effectively and non-invasively estimate the live weight of Anadolu-T broilers based on simple morphometric traits.

All tested ML algorithms achieved excellent predictive performance (R^2^ > 0.98), confirming that back length and width provide sufficient biometric information for accurate live weight estimation. The KNN algorithm produced the most stable and accurate results across male, female, and mixed datasets, showing the lowest overall prediction errors. Ensemble models such as RF and XGB achieved comparable accuracy to KNN, demonstrating strong capability in modeling nonlinear morphometric relationships.

The log-transformed regression models based on body surface pixel area achieved high accuracy confirming that digital image analysis can serve as a reliable alternative to manual weighing. Weekly RMSE and MAPE trends showed decreasing proportional errors toward later growth stages, indicating that the models effectively adapted to the dynamic growth of broilers.

The integration of ML algorithms with image-based morphometric analysis offers a practical, cost-effective, and non-invasive method for real-time weight estimation in broiler production. Future research should focus on expanding datasets to multiple genotypes and rearing environments, automating image capture, and integrating environmental or behavioral data to enhance scalability and robustness.

## Figures and Tables

**Table 1:** Descriptive summary of variables used in model training and testing.

Parameters	Training			Testing		
	Length (mm)	Width (mm)	Live Weight (g)	Length (mm)	Width (mm)	Live Weight (g)
	Male					
Min	34.60	30.20	33.10	51.20	28.60	34.90
Max	219.30	169.00	3414.00	213.50	262.70	3162.00
Mean	142.04	103.64	1039.24	142.21	104.23	1066.52
SE	1.35	1.16	24.84	2.11	1.85	40.08
SD	47.01	40.15	861.75	48.01	41.95	910.35
Sk	−0.39	−0.11	0.61	−0.29	0.04	0.57
Kr	−1.14	−1.21	−0.87	−1.26	−1.01	−1.06
	**Female**					
Min	42.70	31.50	33.17	50.90	32.00	38.70
Max	218.90	167.00	2987.00	216.30	169.30	3085.00
Mean	143.72	104.19	1018.36	145.52	106.24	1070.9
SE	1.31	1.14	23.50	2.08	1.79	36.48
SD	44.76	38.80	799.91	46.43	39.92	814.14
Sk	−0.38	−0.08	0.52	−0.5	−0.21	0.38
Kr	−1.07	−1.23	−1.01	−1.08	−1.24	−1.14
	**Mixed (Male and Female)**					
Min	34.60	30.30	33.10	50.90	28.60	36.20
Max	219.30	262.70	3414.00	218.90	166.50	3302.00
Mean	142.72	104.08	1037.53	144.16	104.82	1048.79
SE	0.96	0.83	17.38	1.43	1.24	26.20
SD	46.59	40.14	845.05	45.67	39.46	834.30
Sk	−0.38	−0.07	0.56	−0.42	−0.13	0.54
Kr	−1.15	−1.17	−0.96	−1.08	−1.21	−0.95

Min: minimum, Max: maximum, Mean: mean, SD: standard deviation, Sk: skewness, Kr: kurtosis.

**Table 2:** Best-tuned hyperparameter values for the ML algorithms used in model development.

Algorithms	Dataset	Hyperparameters
RF	Female	max_depth: 5, min_samples_leaf: 1, min_samples_split: 5, n_estimators: 150
	Male	max_depth: 5, min_samples_leaf: 2, min_samples_split: 5, n_estimators: 100
	Mixed	max_depth: 5, min_samples_leaf: 2, min_samples_split: 2, n_estimators: 150
XGB	Female	learning_rate: 0.05, max_depth: 3, n_estimators: 100, subsample: 1.0
	Male	learning_rate: 0.05, max_depth: 3, n_estimators: 200, subsample: 0.8
	Mixed	learning_rate: 0.05, max_depth: 3, n_estimators: 180, subsample: 1.0
SVR	Female	regularization parameter (C): 1000, epsilon (ε): 0.01, kernel: rbf
	Male	regularization parameter (C): 1000, epsilon (ε): 0.5, kernel: rbf
	Mixed	regularization parameter (C): 1000, epsilon (ε): 0.01, kernel: rbf
KNN	Female	n_neighbors: 11, power parameter: 1, weights: uniform
	Male	n_neighbors: 13, power parameter: 1, weights: uniform
	Mixed	n_neighbors: 13, power parameter: 1, weights: uniform

**Table 3:** Model evaluation results for male, female, and mixed broiler dataset.

Data	Algorithms	Training			Testing		
		R^2^	RMSE (g)	MAPE (%)	R^2^	RMSE (g)	MAPE (%)
Male	RF	0.989	91.747	8.132	0.985	111.274	9.112
	XGB	0.991	83.056	7.769	0.984	113.252	9.049
	SVR	0.985	104.937	8.250	0.983	117.987	8.459
	KNN	0.987	98.531	7.543	0.985	111.709	8.366
	MLR	0.946	200.341	15.037	0.930	241.435	15.412
Female	RF	0.989	84.599	7.592	0.984	102.471	8.848
	XGB	0.990	81.362	6.707	0.984	103.081	8.012
	SVR	0.984	100.365	7.503	0.984	103.446	7.778
	KNN	0.986	93.146	7.092	0.984	102.678	7.797
	MLR	0.942	193.348	12.918	0.940	199.012	13.377
Mixed	RF	0.987	98.068	8.268	0.983	107.378	8.853
	XGB	0.986	98.349	8.669	0.984	106.688	9.133
	SVR	0.983	108.785	8.019	0.983	109.233	8.171
	KNN	0.986	100.542	7.510	0.982	111.509	8.205
	MLR	0.948	193.154	14.066	0.942	200.305	13.909

**Table 4:** Regression coefficients and performance statistics for log-transformed image-based models developed separately for male, female, and mixed datasets.

Data	Model	Training			Testing		
		R^2^	RMSE (g)	MAPE (%)	R^2^	RMSE (g)	MAPE (%)
Male	$\begin{array}{cc} LogLW=-4.127 & +\;(0.124\times Day)\\ & \;+\;(1.261\times logP)\\ & \;-\;(0.020\times Day\times logP) \end{array}$ $SE={\left(0.094\right)}^{**}\;{\left(0.003\right)}^{**}\;{\left(0.021\right)}^{**}\;{\left(0.001\right)}^{**}$	0.987	94.472	7.510	0.990	95.601	7.736
Female	$\begin{array}{cc} LogLW=-4.380 & +\;(0.127\times Day)\\ & \;+\;(1.318\times logP)\\ & \;-\;(0.021\times Day\times logP) \end{array}$ $SE={\left(0.079\right)}^{**}\;{\left(0.002\right)}^{**}\;{\left(0.017\right)}^{**}\;{\left(0.001\right)}^{**}$	0.987	89.404	6.701	0.988	92.276	6.816
Mixed	$\begin{array}{cc} LogLW=-4.294 & +\;(0.124\times Day)\\ & \;+\;(1.299\times logP)\\ & \;-\;(0.021\times Day\times logP) \end{array}$ $SE={\left(0.061\right)}^{**}\;{\left(0.002\right)}^{**}\;{\left(0.014\right)}^{**}\;{\left(0.001\right)}^{**}$	0.986	96.143	7.156	0.989	101.197	7.266

Log LW: Log-transformed live weight, Log P: Log-transformed body surface pixel number, SE: Standard error, ** (*p* < 0.01).

**Table 5:** Weekly predictive performance of the log-transformed image-based regression model for male, female, and mixed datasets.

	Days	RMSE (g)	MAPE (%)
Male	1–7	6.926	8.581
	8–14	24.981	8.726
	15–21	55.692	8.355
	22–28	99.874	7.310
	29–35	130.224	6.204
	36–42	157.001	5.109
Female	1–7	7.694	8.053
	8–14	22.077	7.690
	15–21	52.198	7.838
	22–28	79.377	6.249
	29–35	115.564	5.368
	36–42	162.010	5.524
Mixed	1–7	7.303	8.423
	8–14	23.630	8.264
	15–21	54.272	8.199
	22–28	89.520	6.726
	29–35	123.524	5.817
	36–42	173.447	5.751

## Data Availability

The data presented in this study are available on request from the corresponding author.
